# The combined effect of smoking tobacco and drinking alcohol on cause-specific mortality: a 30 year cohort study

**DOI:** 10.1186/1471-2458-10-789

**Published:** 2010-12-24

**Authors:** Carole L Hart, George Davey Smith, Laurence Gruer, Graham CM Watt

**Affiliations:** 1 Centre for Population & Health Sciences, College of Medical, Veterinary and Life Sciences, University of Glasgow, Public Health & Health Policy, 1 Lilybank Gardens, Glasgow G12 8RZ, UK; 2 School of Social and Community Medicine, University of Bristol, Oakfield House, Oakfield Grove, Bristol BS8 2BN, UK; 3 NHS Health Scotland, Elphinstone House, 65 West Regent Street, Glasgow G2 2AF, UK; 4 Centre for Population & Health Sciences, College of Medical, Veterinary and Life Sciences, University of Glasgow, General Practice & Primary Care, 1 Horselethill Road, Glasgow G12 9LX, UK

## Abstract

**Background:**

Smoking and consuming alcohol are both related to increased mortality risk. Their combined effects on cause-specific mortality were investigated in a prospective cohort study.

**Methods:**

Participants were 5771 men aged 35-64, recruited during 1970-73 from various workplaces in Scotland. Data were obtained from a questionnaire and a screening examination. Causes of death were all cause, coronary heart disease (CHD), stroke, alcohol-related, respiratory and smoking-related cancer. Participants were divided into nine groups according to their smoking status (never, ex or current) and reported weekly drinking (none, 1-14 units and 15 or more). Cox proportional hazards models were used to obtain relative rates of mortality, adjusted for age and other risk factors.

**Results:**

In 30 years of follow-up, 3083 men (53.4%) died. Compared with never smokers who did not drink, men who both smoked and drank 15+ units/week had the highest all-cause mortality (relative rate = 2.71 (95% confidence interval 2.31-3.19)). Relative rates for CHD mortality were high for current smokers, with a possible protective effect of some alcohol consumption in never smokers. Stroke mortality increased with both smoking and alcohol consumption. Smoking affected respiratory mortality with little effect of alcohol. Adjusting for a wide range of confounders attenuated the relative rates but the effects of alcohol and smoking still remained. Premature mortality was particularly high in smokers who drank 15 or more units, with a quarter of the men not surviving to age 65. 30% of men with manual occupations both smoked and drank 15+ units/week compared with only 13% with non-manual ones.

**Conclusions:**

Smoking and drinking 15+ units/week was the riskiest behaviour for all causes of death.

## Background

Numerous studies have shown the serious adverse effects of lifelong tobacco smoking, with countless more showing increased mortality rates from chronic heavy drinking. Regular light drinking appears to have little effect on overall mortality and may be protective against coronary heart disease[1]. In reality, many people both drink and smoke[2]. The neurochemical mechanisms of action of nicotine and alcohol appear to be mutually reinforcing[3]. Drinking and smoking together is strongly socially patterned[4], being normative behaviour in pubs, bars and clubs worldwide until the recent introduction in some countries of smoking restrictions in public places[5]. Despite this, few studies have examined the combined effects of smoking and drinking on mortality[6-9]. In this paper, we studied cause specific mortality in 5771 men whose reported smoking and drinking behaviours were recorded at initial screening and who have been followed up for 30 years.

## Methods

The Collaborative study was conducted between 1970 and 1973 on 6022 employed men and 1006 employed women from a variety of 27 factories and other workplaces in Scotland[10]. The response rate was 70% for the workplaces for which response rates were available (87% of the sample). The participants completed a questionnaire and attended a clinic for a physical examination. On the questionnaire, participants reported their occupation and also their father's occupation, from which social class and father's social class, according to the contemporaneous Classification of Occupations, were obtained[11]. They reported the number of siblings[12], age finishing whole-time education and whether the participant was a regular car driver. Postcode of residence was used to obtain the Carstairs deprivation category, based on four census variables[13]. The categories range from 1 (most affluent) to 7 (most deprived). Bronchitis was derived from the MRC questionnaire[14] and angina from the Rose angina questionnaire[15]. Smoking habit was classified as never smoker, ex-smoker or current smoker. Pipe or cigar smokers only were included with the current smokers. Ex-smokers had given up smoking for at least a year, otherwise they were classified as current smokers. This was defined by the study team in 1970 and is used in this paper for stability or comparison purposes. Current and ex-smokers also reported number of cigarettes smoked per day (with ex-smokers reporting the daily amount smoked for as long as a year), whether they inhaled, at what age they started smoking and ex-smokers reported their age when they gave up smoking. Years of smoking were calculated to date of screening. Participants reported the quantity of spirits, beer and wine usually consumed per week. The answers were converted to units of alcohol assuming a measure of spirit to be one unit, a pint of beer to be two units and a bottle of wine to be six units, the appropriate conversions at the time[16]. Alcohol consumption was classified as none, 1-14 units per week and 15 or more units per week. Participants were allocated to one of nine groups, depending on both their alcohol consumption (none, 1-14 units/week, 15+ units/week) and smoking habit (never, ex, current).

At the screening examination, blood pressure was recorded, height and weight were measured, enabling body mass index (BMI) to be calculated in kg/m^2^, an electrocardiogram was taken and plasma cholesterol and forced expiratory volume in one second (FEV1) were measured[10]. Ischaemia on electrocardiogram was defined as any of Minnesota codes 1.1-1.3, 4.1-4.4, 5.1-5.3 and 7[17]. Percent predicted FEV1 was defined as the actual FEV1 as a percentage of the expected FEV1. The expected FEV1 was obtained from linear regressions of age and height, derived from a healthy subset of the population [18].

Participants were flagged at the NHS Central Register, ensuring the study team is informed when members embark (leave the UK) or die. Date and cause of death are provided and deaths occurring in the 30 years after screening were used. Causes of death were grouped as all cause, coronary heart disease (CHD), stroke, alcohol-related, respiratory and smoking-related cancer. Details and ICD 9 and 10 codes of the causes of death are shown in Table 1.

**Table 1:** ICD codes for the causes of death

Cause	ICD 9 codes	ICD 10 codes
Coronary heart disease	410-414	I20-I25
Stroke	430-438	I60-I69, G45
Alcohol-related causes*	141, 143-6, 148-9, 150, 155, 161, 291, 303, 571, E800-E999	C01-C06, C10, C13-C15, C22, C32, F10, K70, K74.6, S00-Y98
Respiratory diseases	460-519	J00- J99
Smoking-related cancer†	140-151, 155, 157, 160-3, 188-9, 205	C00-C16, C22, C25, C30-34, C38.4, C64-C68, C92

* Alcohol-related causes: cancers of the mouth area (excluding lip, salivary glands and nasopharynx), oesophagus, liver or larynx; alcoholic psychoses; alcohol dependence syndrome; chronic liver disease and cirrhosis; and all external causes

† Smoking-related cancer: cancers of the lip, oral cavity and pharynx; oesophagus; stomach; liver; pancreas; nasal cavities, middle ear and accessory sinuses; larynx; trachea, bronchus and lung; pleura; bladder; kidney; and myeloid leukaemia

The current study used men aged between 35 and 64 years at screening. There were 243 men outside this age range. Women were not included due to lack of events and small numbers of women. There were 6 men who were lost to follow-up and were excluded from the study. Smoking data were missing for two men. The analyses were thus conducted on 5771 men with complete data. The 42 men who had embarked were included in analyses to the date of their embarkation.

Cox proportional hazards models were used to obtain relative rates of mortality by smoking and alcohol consumption category, with the proportionality assumption being tested by inspection of Schoenfeld residuals. Never smokers who reported drinking no alcohol were taken as the reference group. The models were adjusted firstly for age, and then for other risk factors. For the risk factor adjustment, the small numbers of missing values of cholesterol (40), BMI (1), percent predicted FEV1 (5) and father's social class (112) were imputed using the means for continuous variables or modes for categorical variables. Adjustment was not made for blood pressure levels, due to strong evidence that alcohol intake increases blood pressure and therefore that such adjustment would be for a variable in the causal pathway between alcohol intake and mortality[19]. Interactions by smoking category and alcohol category were tested using likelihood ratio tests. Means and percentages of risk factors were standardised by 5 year age groups. Stata release 10 was used. At the time of the study, ethical permissions were not required.

## Results

The numbers and percentages of men in each alcohol-smoking group are shown in Table 2. Over half the men were smokers at screening and very few of the never smokers drank 15+ units/week. Just over a fifth of the men smoked and drank 15+ units/week.

**Table 2:** Number and percentage of men by smoking and alcohol consumption category

	Smoking status		
	
Drinking status	Never	Ex	Current
None	490 (8.5%)	471 (8.2%)	873 (15.1%)
1-14 units/week	362 (6.3%)	544 (9.4%)	1273 (22.1%)
15+ units/week	164 (2.8%)	380 (6.6%)	1214 (21.0%)

There were some differences in age between the nine groups, with never smokers being younger and ex-smokers being older than current smokers (Table 3). Non-drinkers tended to be older than drinkers. Current smokers who drank 15+ units/week smoked more cigarettes/day than current smokers who drank 1-14 units/week or did not drink. The same pattern was seen for mean cigarettes/day previously smoked by ex-smokers. In 15+ units/week drinkers, there were no differences in mean units/week between smoking groups, although amongst drinkers of 1-14 units/week, current smokers drank more units/week than never or ex-smokers. Nearly 70% of men who smoked and drank 15+ units/week were from manual social classes, compared with 35% of men who had never smoked and either did not drink, or drank 1-14 units/week. Systolic and diastolic blood pressure and BMI generally increased with alcohol consumption, but decreased with smoking, with never smokers who drank 15+ units/week having the highest blood pressures and BMIs.

**Table 3:** Age adjusted risk factors by smoking and alcohol consumption category in men

	Smoking status		
	
Drinking status	Never	Ex	Current
**Mean age**			
None	47.7 (47.0 - 48.3)	50.3 (49.7 - 50.8)	49.1 (48.7 - 49.5)
1-14 units^1^/week	45.4 (44.8 - 46.1)	49.1 (48.6 - 49.6)	48.2 (47.8 - 48.6)
15+ units/week	46.3 (45.3 - 47.3)	49.0 (48.3 - 49.6)	47.5 (47.2 - 47.9)
			
**Mean cigarettes/day**			
None	0	18.3 (17.3 - 19.3)	17.2 (16.6 - 17.8)
1-14 units/week	0	18.6 (17.7 - 19.4)	17.5 (17.0 - 18.0)
15+ units/week	0	21.9 (20.7 - 23.0)	19.4 (18.9 - 19.9)
			
**Mean units/week**			
None	0	0	0
1-14 units/week	6.9 (6.5 - 7.4)	6.9 (6.5 - 7.2)	7.6 (7.4 - 7.8)
15+ units/week	32.2 (29.6 - 34.8)	30.4 (28.6 - 32.1)	30.1 (29.2 - 31.0)
			
**Mean siblings**			
None	3.0 (2.7 - 3.2)	3.1 (2.8 - 3.3)	3.4 (3.3 - 3.6)
1-14 units/week	3.0 (2.7 - 3.2)	3.0 (2.8 - 3.2)	3.4 (3.2 - 3.5)
15+ units/week	3.9 (3.5 - 4.4)	3.6 (3.3 - 3.8)	4.0 (3.9 - 4.2)
			
**Mean plasma cholesterol **(mmol/L)			
None	5.89 (5.80 - 5.98)	5.83 (5.74 - 5.93)	5.84 (5.77 - 5.91)
1-14 units/week	5.85 (5.75 - 5.96)	6.06 (5.97 - 6.15)	5.91 (5.85 - 5.97)
15+ units/week	5.98 (5.81 - 6.15)	6.02 (5.91 - 6.13)	5.76 (5.70 - 5.81)
			
**Mean systolic blood pressure **(mm/Hg)			
None	132.5 (130.9 - 134.0)	133.5 (132.1 - 134.9)	133.0 (132.0 - 134.1)
1-14 units/week	133.0 (131.0 - 135.0)	132.9 (131.6 - 134.3)	132.0 (131.2 - 132.9)
15+ units/week	139.8 (137.1 - 142.6)	138.2 (136.4 - 140.0)	135.7 (134.6 - 136.8)
			
**Mean diastolic blood pressure **(mm/Hg)			
None	83.6 (82.7 - 84.5)	84.5 (83.6 - 85.5)	82.5 (81.9 - 83.2)
1-14 units/week	84.7 (83.5 - 86.0)	84.5 (83.6 - 85.3)	82.1 (81.6 - 82.7)
15+ units/week	89.0 (87.2 - 90.8)	87.6 (86.5 - 88.6)	84.0 (83.5 - 84.6)
			
**Mean body mass index (kg/m^2^)**			
None	25.6 (25.3 - 25.9)	25.5 (25.3 - 25.8)	24.9 (24.7 - 25.1)
1-14 units/week	25.6 (25.3 - 25.9)	25.5 (25.3 - 25.8)	24.7 (24.6 - 24.9)
15+ units/week	27.4 (26.9 - 27.9)	26.0 (25.7 - 26.3)	24.8 (24.6 - 24.9)
			
**Mean % predicted FEV1^2 ^**(%)			
None	99.9 (98.2 - 101.6)	99.2 (97.3 - 101.0)	91.9 (90.6 - 93.2)
1-14 units/week	99.1 (96.7 - 101.6)	96.5 (94.7 - 98.2)	93.3 (92.3 - 94.4)
15+ units/week	94.3 (91.1 - 97.5)	96.1 (93.9 - 98.2)	89.6 (88.5 - 90.7)
			
**% manual social class**			
None	35.2 (30.9 - 39.4)	36.5 (32.0 - 40.9)	46.8 (43.5 - 50.1)
1-14 units/week	35.3 (30.0 - 40.6)	35.6 (31.6 - 39.6)	46.8 (44.1 - 49.6)
15+ units/week	66.6 (59.2 - 73.9)	56.1 (51.2 - 61.1)	69.9 (67.4 - 72.5)
			
**% father's manual social class**			
None	71.3 (67.2 - 75.4)	70.2 (65.8 - 74.6)	78.7 (75.9 - 81.4)
1-14 units/week	73.2 (68.2 - 78.2)	64.4 (60.3 - 68.5)	72.1 (69.6 - 74.6)
15+ units/week	83.4 (77.6 - 89.3)	77.5 (73.4 - 81.7)	84.2 (82.1 - 86.3)
			
**% left education ≤ 14 yrs**			
None	41.8 (37.4 - 46.1)	46.3 (41.9 - 50.8)	51.6 (48.4 - 54.7)
1-14 units/week	39.3 (33.9 - 44.7)	38.1 (34.2 - 42.0)	46.5 (43.9 - 49.2)
15+ units/week	61.9 (54.5 - 69.3)	56.8 (51.9 - 61.7)	66.2 (63.7 - 68.7)
			
**% depcat 6 & 7^3^**			
None	22.4 (18.6 - 26.1)	19.1 (15.5 - 22.8)	28.1 (25.1 - 31.0)
1-14 units/week	22.7 (18.0 - 27.4)	17.4 (14.2 - 20.6)	27.1 (24.7 - 29.6)
15+ units/week	41.0 (33.3 - 48.7)	31.7 (27.0 - 36.4)	40.5 (37.8 - 43.3)
			
**% car user**			
None	54.4 (49.9 - 58.8)	56.4 (51.8 - 61.1)	53.2 (49.9 - 56.6)
1-14 units/week	64.2 (58.8 - 69.5)	68.6 (64.6 - 72.5)	54.3 (51.6 - 57.0)
15+ units/week	41.7 (34.0 - 49.3)	52.5 (47.5 - 57.5)	34.1 (31.5 - 36.7)
			
**% angina**			
None	5.8 (3.7 - 7.9)	4.2 (2.5 - 5.8)	7.0 (5.3 - 8.7)
1-14 units/week	1.0 (-0.05 to 2.1)	5.0 (3.2 - 6.8)	6.7 (5.3 - 8.1)
15+ units/week	1.5 (-0.2 to 3.2)	5.6 (3.3 - 7.9)	8.5 (6.9 - 10.1)
			
**% with ischaemia on ECG^4^**			
None	4.3 (2.5 - 6.1)	7.2 (4.9 - 9.5)	4.8 (3.5 - 6.2)
1-14 units/week	6.1 (3.2 - 8.9)	7.1 (4.9 - 9.3)	5.1 (3.9 - 6.3)
15+ units/week	3.8 (1.0 - 6.5)	7.2 (4.6 - 9.8)	5.9 (4.6 - 7.2)
			
**% bronchitis**			
None	0.4 (-0.2 to 1.0)	0.8 (0.1 - 1.4)	2.3 (1.3 - 3.4)
1-14 units/week	0	1.5 (0.5 - 2.5)	2.3 (1.5 - 3.1)
15+ units/week	1.1 (-0.4 to 2.7)	1.8 (0.5 - 3.1)	4.4 (3.2 - 5.5)
			

^1 ^1 unit = 1 measure of spirits, or half a pint of beer or a sixth of a bottle of wine

^2 ^FEV1 = forced expiratory volume in 1 second

^3 ^Depcat 6&7 = the 2 most deprived deprivation categories

^4 ^ECG = electrocardiogram

**Figure 1:**
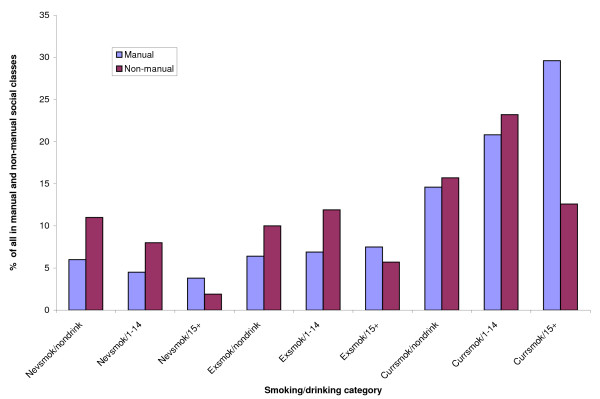
**Comparison of % of non-manual and manual social class men in each smoking/drinking category**.

Table 4 and Figure 1 show the percentages of men in manual and non-manual social classes who were in each smoking and drinking category. They show that men in manual social classes were much more likely to be smokers and heavy drinkers and less likely to be non-smokers who drank moderately or not at all.

**Table 4:** Number and percentage of manual and of non-manual men in each smoking and drinking category

	Smoking status		
	
Drinking status	Never	Ex	Current
None			
Manual	170 (6%)	181 (6.4%)	415 (14.6%)
Non manual	319 (11%)	290 (10%)	457 (15.7%)
1-14 units/week			
Manual	129 (4.5%)	196 (6.9%)	593 (20.8%)
Non manual	232 (8%)	347 (11.9%)	675 (23.2%)
15+ units/week			
Manual	108 (3.8%)	213 (7.5%)	845 (29.6%)
Non manual	56 (1.9%)	167 (5.7%)	367 (12.6%)

The percentage of men who did not survive to age 65 was high in the current smokers and in the drinkers of 15+ units/week (Table 5). A quarter of the men who both smoked and drank 15+ units/week did not reach their 65^th ^birthday. In the 30 year follow-up period, 3083 men (53.4%) died. Men who both smoked and drank 15+ units/week had the highest age adjusted relative rate of all cause mortality (2.71 (95% CI 2.31, 3.19)) compared with the never smokers who did not drink (Table 6). This group also had the highest relative rate for all the other causes examined. Within each alcohol group, current smokers had the highest age adjusted relative rate for all cause mortality and ex-smokers had higher relative rates than never smokers. Adjustment for other risk factors attenuated the relative rates but the pattern remained. Within each smoking group, there was little effect of alcohol consumption, except in the current smokers where the age adjusted relative rate was raised for the 15+ units/week drinkers. After adjustment for other risk factors, the patterns were similar. Relative rates for CHD mortality were high for current smokers, even after adjustment for risk factors. There was a suggestion of a protective effect of some alcohol consumption on CHD mortality especially amongst the never smokers. Stroke mortality increased with both smoking and alcohol consumption, with current smokers who drank 15+ units/week having an adjusted relative rate of 3.1 (95% CI 1.82, 5.28). For respiratory mortality, there was a clear effect of smoking, but little effect of drinking. Adjustment for risk factors attenuated the relative rates considerably for current smokers. Ex-smokers had noticeably high relative rates of respiratory mortality compared to never smokers. Adjustments using additional socioeconomic variables (social class, education, number of siblings, car and deprivation category) gave similar results. There were no significant interactions seen between smoking and drinking for any of the causes of death (all cause p = 0.85, CHD p = 0.39, stroke p = 0.91, alcohol-related p = 0.47, respiratory p = 0.44 and smoking-related cancer p = 0.72). The analyses in Table 6 were repeated without adjusting for BMI or for cholesterol, in case these variables were on the causal pathway between smoking or alcohol and mortality (Additional file 1: Table S1). Although there were some slight differences in effect size, the results were similar overall. The mortality analyses were also repeated excluding deaths occurring in the first 5 years of follow-up. Results were generally similar, with the same overall pattern (Additional file 1: Table S2 and S3).

**Table 5:** Percentage of men not surviving to age 65 by smoking and alcohol consumption category

	Smoking Status		
	
Drinking Status	Never	Ex	Current
None	7.3%	13.2%	20.5%
1-14 units/week	7.7%	12.9%	19.3%
15+ units/week	12.8%	15.0%	25.3%

**Table 6:** Relative rates of mortality in 30 years of follow-up by smoking and alcohol consumption category

	Smoking status		
	
Drinking status	Never	Ex	Current
**All cause deaths**			
None			
No of deaths	181	257	540
RR_1_	1*	1.43 (1.18 - 1.73)	2.10 (1.78 - 2.49)
RR_2_	1	1.38 (1.14 - 1.67)	1.99 (1.68 - 2.35)
1-14 units/week			
No of deaths	100	250	707
RR_1_	0.87 (0.68 - 1.10)	1.19 (0.98 - 1.44)	1.93 (1.64 - 2.27)
RR_2_	0.86 (0.68 - 1.10)	1.14 (0.94 - 1.38)	1.88 (1.59 - 2.21)
15+ units/week			
No of deaths	59	203	786
RR_1_	1.15 (0.86 - 1.54)	1.68 (1.37 - 2.05)	2.71 (2.31 - 3.19)
RR_2_	1.06 (0.79 - 1.42)	1.56 (1.27 - 1.90)	2.50 (2.12 - 2.94)
			
**Coronary heart disease deaths**			
None			
No of deaths	76	92	196
RR_1_	1	1.21 (0.89 - 1.64)	1.75 (1.34 - 2.28)
RR_2_	1	1.13 (0.83 - 1.53)	1.68 (1.28 - 2.19)
1-14 units/week			
No of deaths	36	85	257
RR_1_	0.74 (0.49 - 1.09)	0.96 (0.71 - 1.31)	1.61 (1.25 - 2.08)
RR_2_	0.73 (0.49 - 1.09)	0.88 (0.64 - 1.20)	1.60 (1.23 - 2.06)
15+ units/week			
No of deaths	14	69	251
RR_1_	0.64 (0.36 - 1.13)	1.32 (0.95 - 1.83)	1.94 (1.50 - 2.51)
RR_2_	0.54 (0.30 - 0.95)	1.14 (0.82 - 1.58)	1.82 (1.40 - 2.36)
			
**Stroke deaths**			
None			
No of deaths	17	23	48
RR_1_	1	1.31 (0.70 - 2.45)	2.16 (1.24 - 3.75)
RR_2_	1	1.26 (0.67 - 2.37)	2.10 (1.20 - 3.67)
1-14 units/week			
No of deaths	13	23	66
RR_1_	1.30 (0.63 - 2.68)	1.14 (0.61 - 2.14)	2.11 (1.24 - 3.60)
RR_2_	1.32 (0.64 - 2.73)	1.15 (0.61 - 2.16)	2.16 (1.26 - 3.69)
15+ units/week			
No of deaths	9	22	75
RR_1_	2.06 (0.92 - 4.63)	2.05 (1.09 - 3.86)	3.26 (1.92 - 5.53)
RR_2_	2.04 (0.90 - 4.59)	1.98 (1.05 - 3.74)	3.10 (1.82 - 5.28)
			
**Alcohol-related deaths**			
None			
No of deaths	5	8	16
RR_1_	1	1.72 (0.56 - 5.28)	2.18 (0.80 - 5.94)
RR_2_	1	1.72 (0.56 - 5.27)	2.10 (0.77 - 5.73)
1-14 units/week			
No of deaths	9	14	24
RR_1_	2.55 (0.85 - 7.62)	2.52 (0.91 - 6.99)	2.22 (0.85 - 5.82)
RR_2_	2.53 (0.85 - 7.57)	2.58 (0.93 - 7.17)	2.21 (0.84 - 5.81)
15+ units/week			
No of deaths	7	16	68
RR_1_	4.51 (1.43 - 14.2)	4.69 (1.72 - 12.8)	7.45 (3.0 - 18.5)
RR_2_	4.23 (1.34 - 13.4)	4.54 (1.66 - 12.4)	6.98 (2.80 - 17.4)
			
**Respiratory deaths**			
None			
No of deaths	5	20	59
RR_1_	1	3.91 (1.47 - 10.4)	9.15 (3.67 - 22.8)
RR_2_	1	3.62 (1.36 - 9.65)	6.80 (2.72 - 17.0)
1-14 units/week			
No of deaths	5	19	65
RR_1_	1.70 (0.49 - 5.87)	3.21 (1.20 - 8.60)	7.15 (2.88 - 17.8)
RR_2_	1.76 (0.51 - 6.11)	3.23 (1.20 - 8.66)	5.71 (2.30 - 14.2)
15+ units/week			
No of deaths	4	10	77
RR_1_	3.12 (0.84 - 11.6)	3.20 (1.09 - 9.37)	11.6 (4.68 - 28.7)
RR_2_	3.84 (1.03 - 14.4)	3.26 (1.11 - 9.55)	8.28 (3.33 - 20.6)
			
**Smoking-related cancer deaths**			
None			
No of deaths	15	34	111
RR_1_	1	2.31 (1.26 - 4.25)	5.10 (2.97 - 8.74)
RR_2_	1	2.31 (1.26 - 4.24)	4.83 (2.81 - 8.30)
1-14 units/week			
No of deaths	8	39	152
RR_1_	0.81 (0.34 - 1.91)	2.26 (1.25 - 4.10)	4.86 (2.86 - 8.26)
RR_2_	0.81 (0.34 - 1.92)	2.32 (1.28 - 4.21)	4.79 (2.82 - 8.16)
15+ units/week			
No of deaths	7	24	180
RR_1_	1.60 (0.65 - 3.92)	2.36 (1.24 - 4.49)	7.10 (4.19 - 12.0)
RR_2_	1.55 (0.63 - 3.80)	2.33 (1.22 - 4.44)	6.55 (3.86 - 11.1)

* 1 is reference, 95% confidence intervals in parentheses

RR_1 _Relative rate adjusted for age

RR_2 _Relative rate adjusted for age, cholesterol, body mass index, % predicted FEV1, father's social class, angina, ECG ischaemia and bronchitis

1 unit = 1 measure of spirits, or half a pint of beer or a sixth of a bottle of wine

Men only

The mortality analyses were repeated using 4597 men who were current or ex-smokers who had complete data on details of their smoking habit. The relative rates were additionally adjusted for the number of cigarettes smoked per day, whether inhaled or not, the age started smoking and the years smoked to screening. These extra adjustments had only a small effect (Additional file 1: Table S4 and S5).

## Discussion

Smoking and drinking 15+ units/week was the riskiest behaviour for all the causes of death considered in this study. Men in this category smoked more cigarettes/day than current smokers in the other drinking categories, suggesting that excess drinking and heavy smoking occur together, and the extra number of cigarettes/day may be contributing to the excess mortality. Smoking had stronger effects than alcohol for most of the causes investigated. Current smokers had consistently high mortality rates. Ex-smokers had lower mortality than current smokers, showing the beneficial effects of smoking cessation, but they had higher mortality than never smokers, especially due to respiratory disease and smoking-related cancer. This demonstrates the long-lasting effects of smoking, even after stopping. We have previously shown that consuming 15 units or more per week of alcohol was associated with increased mortality from several causes[20]. We now show that both smoking and drinking 15 or more units/week increased mortality from some of the above causes and also smoking-related cancer. For the specific causes, there was a protective effect of alcohol seen in CHD mortality, particularly for never smokers, but for stroke mortality, both smoking and alcohol were important. Adjusting for a wide range of confounders attenuated the relative rates but the effects of alcohol and smoking still remained. There could also have been residual confounding due to unmeasured variables such as diet. The interaction terms were not found to be significant: this could have been due to lack of power rather than to no effect. The numbers of deaths from stroke, alcohol-related and respiratory causes were small for never smokers, and may have had insufficient power to detect associations in this cohort.

Lower socioeconomic position and low educational attainment were strongly related to both alcohol consumption and smoking in this cohort. For example, 30% of the manual men were both smokers and heavy drinkers compared with only 13% of the non-manual men. On the other hand, only 11% of the manual men were never smokers who drank less than 15 units per week or not at all, compared with 19% of the non-manual men. Given the increased mortality rates associated with both smoking and heavy drinking, this will inevitably contribute to socioeconomic health inequalities. We also recently showed that in this cohort a combination of heavy drinking and obesity had a supra-additive impact on mortality from liver disease[21], further underlining the consequences of multiple risk factors and their potential effect on health inequalities if they are socially patterned. A study of 16,980 men and women in the Netherlands in 1991-8 showed much lower prevalences of smoking and excessive alcohol consumption but similar socioeconomic differences[22]. Smoking and excessive alcohol consumption ranged from 3.5% in the highest educated men and women to 6.1% in the lowest. In a study of 22,457 middle-aged men and women from Norfolk, United Kingdom in 1993-7, there were more current smokers in lower social classes but the proportion drinking 14 or more units of alcohol per week was greater in higher social classes, unlike the current study[23]. A study of tobacco and hazardous or harmful alcohol use in 39,290 participants from Thailand in 2004 found the strongest predictor of harmful or hazardous alcohol consumption was smoking and the strongest predictor of smoking was harmful or hazardous alcohol use in both men and women[24]. That study found no relationship between both smoking and excess alcohol consumption with education, but suggested that men in middle income groups were more likely to both smoke and use excess alcohol. The message here seems to be that social patterning of drinking and smoking is culturally specific. Other than recognising the adverse consequences of combining both, their social patterning cannot be generalised from one country or even one region to another.

There have been other studies of the effects of smoking and alcohol consumption on mortality, but they did not have such a long follow-up as in the current study, nor were they able to investigate the effects on as many different causes of death.

Our results for all cause mortality were similar to those from a large study of 18,244 middle-aged Chinese men, screened in1986-9, although the follow-up time was only 6.7 years on average and smoking and alcohol definitions were different[6]. Within each drinking category, risk of death was higher for smokers and increased with amount smoked. Within each smoking category, there was a suggestion that drinkers of 1-28 drinks/week had a lower risk of death than non-drinkers or heavy drinkers. The highest risk was seen in the heaviest drinkers who also smoked. A 1986 study of 30,518 women aged 55-69 in the United States, with 14 years of follow-up analysed the relationship between alcohol and mortality and cancer incidence by each smoking category separately[7]. Alcohol consumption was inversely associated with all cause and CHD mortality for never and ex-smokers, and was positively associated with cancer incidence for current and ex-smokers. There were no clear associations with cancer mortality. Attention was drawn to the lack of a protective effect of alcohol on CHD mortality in smokers: this was also observed in the current study. A study of 64,515 Chinese men aged from 30 to 89 screened between 1996 and 2000, with an average of 4.6 years of follow-up related alcohol and smoking to mortality in a similar way to the current study[8]. The highest all cause mortality was seen in the heavy drinkers and heavy smokers. There was a protective effect of moderate drinking for all cause and cardiovascular disease (CVD) mortality which was stronger in non-smokers than ever smokers. Heavy drinking was associated with increased cancer mortality and there was no protective effect seen in moderate drinking for cancer mortality, even in the non-smokers.

Other studies have looked at incidence of disease rather than mortality. In a study of stroke incidence in 1991-2 of 45,449 Swedish women aged 30-50, with an average of 11 years of follow-up, current smokers who did not drink alcohol had a four-fold increased risk of stroke compared with never smokers who did not drink[9]. Current smokers who did drink alcohol had lower relative risks than current smokers who did not drink alcohol; however, the numbers of women and cases of stroke were low in the higher alcohol category (≥ 70 g/week) in that study (3,793 women with 17 stroke cases), raising the possibility that an adverse effect of heavy drinking could have been missed. There was some evidence of a protective effect of moderate drinking in never smokers. No such protective effect of alcohol was seen with stroke mortality in the current study, but this could be because they were older, were men and stroke mortality rather than incidence was being investigated. A pooled analysis found positive relationships between alcohol and lung cancer among non-smoking men only although the absolute risk of lung cancer was small in that group[25]. There were no relationships seen for smokers, former smokers or non-smoking women. In a large study of women in 1996-2001, increasing alcohol intake was strongly associated with the incidence of upper aerodigestive tract cancers in current smokers, but not in never or former smokers[26].

The current study showed that alcohol and smoking both contribute to mortality risk. However this may not necessarily be the case for all causes. A large study of UK women in 1996-2001 showed that alcohol consumption reduced, but smoking increased the risk of gallbladder disease death or hospital admission[27]. That study also showed large effects of both smoking and alcohol on death or hospital admission for cirrhosis[27].

### Strengths

The strengths of the study were the long follow-up, which was almost complete, and the ability to adjust for several risk factors and look at several causes of death. For example, unlike in other studies, CVD was broken down into CHD and stroke. This is important as alcohol consumption can have very different effects on these two diseases. We were also able to keep ex-smokers as a separate group, whereas other studies combined ex and current smokers to form an ever smoking group.

### Limitations

In common with other longitudinal studies, alcohol consumption was self-reported and may thus have been underestimated. We have previously reported on the reliability of the alcohol data from the present cohort[16]. Former drinkers were classified as non-drinkers, as we could not differentiate between former drinkers and never drinkers. Former drinkers may be unhealthier than the other non-drinkers if they had given up due to poor health, which could increase the mortality risk in the non-drinkers. However, this cohort of working men would be expected to be healthier than other cohorts so we would expect fewer former drinkers among the non-drinkers. As alcohol and smoking were reported at screening, we do not know if these practices were continued or changed during follow-up. If some ex-smokers took up smoking again, it would have the effect of increasing mortality in the ex-smokers. Also some current smokers at screening would have subsequently given up, which would decrease the mortality in the current smokers. Our main analyses did not take into account the amount of cigarettes smoked in the current smoking group. However there was little effect of additionally adjusting for all the associated smoking habit variables. Although adjustment was made for several covariates, the study did not record others such as dietary intake, family history of disease or adequate information on exercise. The cohort consisted of employed men, who would be healthier on average than men from general populations, so the results may underestimate absolute risk, but the relative risks are likely to be generalisable to male populations from similar contexts.

### Policy implications

Smokers who also drank 15+ units/week had the highest risk of dying from all the causes compared to the other groups. Drinking 15+ units is lower than the weekly upper limit of 21 units recommended by the UK government for men[28]. As it has been shown previously in this cohort that drinking 15-21 units per week and over leads to an increased risk of mortality[20], the cut-off of 15 was a reasonable choice, although we do not have sufficient data on consumption throughout the follow-up period to recommend changing the weekly limits. Smoking had a greater adverse effect on mortality than alcohol consumption, and ex-smokers who had stopped smoking before the screening examination had lower mortality risks than smokers. These findings reinforce the importance of continuing to prioritise smoking cessation across the whole population. Given the strong links between smoking and heavy drinking, it may also be helpful to devise policies aimed at reducing both smoking and alcohol consumption in population groups where this is common.

## Conclusion

Smoking and drinking 15+ units/week was the riskiest behaviour for all causes of death.

## Competing interests

The authors declare that they have no competing interests.

## Authors' contributions

CH performed the statistical analysis and wrote the first draft of the manuscript. GW, LG, GDS and CH conceived of the study, and participated in its design and coordination and helped to draft the manuscript. All authors read and approved the final manuscript.

## Funding

CH was supported for this study by NHS Health Scotland. The funding body had no role in the study design; in the collection, analysis and interpretation of data; writing of the manuscript; or the decision to submit the manuscript for publication.

## Pre-publication history

The pre-publication history for this paper can be accessed here:

http://www.biomedcentral.com/1471-2458/10/789/prepub

## Supplementary Material

Additional file 1 **Additional tables S1 to S5**. Table S1. Relative rates of mortality in 30 years of follow-up in men by smoking and alcohol consumption category excluding adjustment for body mass index and cholesterol. Table S2. Number and percentage of men by smoking and alcohol consumption category, excluding deaths in 1st 5 years. Table S3. Relative rates of mortality in 30 years of follow-up in men by smoking and alcohol consumption category, excluding deaths in 1st 5 years. Table S4. Number of men by smoking and alcohol consumption category, for current and ex-smokers only. Table S5. Relative rates of mortality by smoking and alcohol consumption category in men for current and ex-smokers only. Click here for file
