# Supplementary material for: The combined effect of smoking tobacco and drinking alcohol on cause-specific mortality: a 30 year cohort study
Source: BMC Public Health. 2010 Dec 24;10:789. doi: 10.1186/1471-2458-10-789 (PMC3022858; doi:10.1186/1471-2458-10-789)
Supplement: Additional file 1 — Additional tables S1 to S5. Table S1. Relative rates of mortality in 30 years of follow-up in men by smoking and alcohol consumption category excluding adjustment for body mass index and cholesterol. Table S2. Number and percentage of men by smoking and alcohol consumption category, excluding deaths in 1st 5 years. Table S3. Relative rates of mortality in 30 years of follow-up in men by smoking and alcohol consumption category, excluding deaths in 1st 5 years. Table S4. Number of men by smoking and alcohol consumption category, for current and ex-smokers only. Table S5. Relative rates of mortality by smoking and alcohol consumption category in men for current and ex-smokers only. [file 1471-2458-10-789-S1.DOC]

# Table S1. Relative rates of mortality in 30 years of follow-up in men by smoking and alcohol consumption category excluding adjustment for body mass index and cholesterol

|  |  | Smoking status |  |
| --- | --- | --- | --- |
| Drinking status | Never | Ex | Current |
|  |  |  |  |
| All cause deaths |  |  |  |
| None |  |  |  |
| No of deaths | 181 | 257 | 540 |
| RR_1_ | 1* | 1.43 (1.18 – 1.73) | 2.10 (1.78 – 2.49) |
| RR_2_ | 1 | 1.38 (1.14 – 1.67) | 1.99 (1.68 – 2.35) |
| RR_3_ | 1 | 1.37 (1.13 – 1.66) | 1.95 (1.64 – 2.31) |
| RR_4_ | 1 | 1.39 (1.15 – 1.69) | 1.99 (1.68 – 2.36) |
| 1-14 units / week |  |  |  |
| No of deaths | 100 | 250 | 707 |
| RR_1_ | 0.87 (0.68 – 1.10) | 1.19 (0.98 – 1.44) | 1.93 (1.64 – 2.27) |
| RR_2_ | 0.86 (0.68 – 1.10) | 1.14 (0.94 – 1.38) | 1.88 (1.59 – 2.21) |
| RR_3_ | 0.86 (0.68 – 1.10) | 1.13 (0.93 – 1.37) | 1.84 (1.56 – 2.17) |
| RR_4_ | 0.87 (0.68 – 1.11) | 1.17 (0.96 – 1.41) | 1.90 (1.61 – 2.24) |
| 15+ units / week |  |  |  |
| No of deaths | 59 | 203 | 786 |
| RR_1_ | 1.15 (0.86 – 1.54) | 1.68 (1.37 – 2.05) | 2.71 (2.31 – 3.19) |
| RR_2_ | 1.06 (0.79 – 1.42) | 1.56 (1.27 – 1.90) | 2.50 (2.12 – 2.94) |
| RR_3_ | 1.10 (0.82 – 1.47) | 1.58 (1.29 – 1.93) | 2.45 (2.08 – 2.89) |
| RR_4_ | 1.07 (0.80 – 1.44) | 1.58 (1.29 – 1.93) | 2.50 (2.12 – 2.94) |
|  |  |  |  |
| Coronary heart disease deaths |  |  |  |
| None |  |  |  |
| No of deaths | 76 | 92 | 196 |
| RR_1_ | 1 | 1.21 (0.89 – 1.64) | 1.75 (1.34 – 2.28) |
| RR_2_ | 1 | 1.13 (0.83 – 1.53) | 1.68 (1.28 – 2.19) |
| RR_3_ | 1 | 1.11 (0.82 – 1.51) | 1.61 (1.23 – 2.10) |
| RR_4_ | 1 | 1.16 (0.86 – 1.58) | 1.70 (1.30 – 2.22) |
| 1-14 units / week |  |  |  |
| No of deaths | 36 | 85 | 257 |
| RR_1_ | 0.74 (0.49 – 1.09) | 0.96 (0.71 – 1.31) | 1.61 (1.25 – 2.08) |
| RR_2_ | 0.73 (0.49 – 1.09) | 0.88 (0.64 – 1.20) | 1.60 (1.23 – 2.06) |
| RR_3_ | 0.73 (0.49 – 1.09) | 0.87 (0.64 – 1.18) | 1.52 (1.18 – 1.97) |
| RR_4_ | 0.74 (0.50 – 1.10) | 0.94 (0.69 – 1.28) | 1.65 (1.27 – 2.13) |
| 15+ units / week |  |  |  |
| No of deaths | 14 | 69 | 251 |
| RR_1_ | 0.64 (0.36 – 1.13) | 1.32 (0.95 – 1.83) | 1.94 (1.50 – 2.51) |
| RR_2_ | 0.54 (0.30 – 0.95) | 1.14 (0.82 – 1.58) | 1.82 (1.40 – 2.36) |
| RR_3_ | 0.59 (0.33 – 1.04) | 1.18 (0.85 – 1.64) | 1.75 (1.35 – 2.27) |
| RR_4_ | 0.57 (0.32 – 1.0) | 1.20 (0.86 – 1.66) | 1.81 (1.40 – 2.35) |

|  |  |  |  |
| --- | --- | --- | --- |
| Stroke deaths |  |  |  |
| None |  |  |  |
| No of deaths | 17 | 23 | 48 |
| RR_1_ | 1 | 1.31 (0.70 – 2.45) | 2.16 (1.24 – 3.75) |
| RR_2_ | 1 | 1.26 (0.67 – 2.37) | 2.10 (1.20 – 3.67) |
| RR_3_ | 1 | 1.25 (0.67 – 2.35) | 2.05 (1.18 – 3.58) |
| RR_4_ | 1 | 1.26 (0.67 – 2.36) | 2.10 (1.20 – 3.66) |
| 1-14 units / week |  |  |  |
| No of deaths | 13 | 23 | 66 |
| RR_1_ | 1.30 (0.63 – 2.68) | 1.14 (0.61 – 2.14) | 2.11 (1.24 – 3.60) |
| RR_2_ | 1.32 (0.64 – 2.73) | 1.15 (0.61 – 2.16) | 2.16 (1.26 – 3.69) |
| RR_3_ | 1.32 (0.64 – 2.73) | 1.15 (0.61 – 2.15) | 2.11 (1.24 – 3.61) |
| RR_4_ | 1.32 (0.64 – 2.72) | 1.14 (0.61 – 2.14) | 2.15 (1.26 – 3.68) |
| 15+ units / week |  |  |  |
| No of deaths | 9 | 22 | 75 |
| RR_1_ | 2.06 (0.92 – 4.63) | 2.05 (1.09 – 3.86) | 3.26 (1.92 – 5.53) |
| RR_2_ | 2.04 (0.90 – 4.59) | 1.98 (1.05 – 3.74) | 3.10 (1.82 – 5.28) |
| RR_3_ | 2.11 (0.94 – 4.74) | 2.0 (1.06 – 3.78) | 3.04 (1.78 – 5.17) |
| RR_4_ | 2.02 (0.90 – 4.55) | 1.97 (1.04 – 3.72) | 3.10 (1.82 – 5.28) |
|  |  |  |  |
| Alcohol-related deaths |  |  |  |
| None |  |  |  |
| No of deaths | 5 | 8 | 16 |
| RR_1_ | 1 | 1.72 (0.56 – 5.28) | 2.18 (0.80 – 5.94) |
| RR_2_ | 1 | 1.72 (0.56 – 5.27) | 2.10 (0.77 – 5.73) |
| RR_3_ | 1 | 1.71 (0.56 – 5.24) | 2.05 (0.75 – 5.60) |
| RR_4_ | 1 | 1.71 (0.56 – 5.23) | 2.08 (0.76 – 5.69) |
| 1-14 units / week |  |  |  |
| No of deaths | 9 | 14 | 24 |
| RR_1_ | 2.55 (0.85 – 7.62) | 2.52 (0.91 – 6.99) | 2.22 (0.85 – 5.82) |
| RR_2_ | 2.53 (0.85 – 7.57) | 2.58 (0.93 – 7.17) | 2.21 (0.84 – 5.81) |
| RR_3_ | 2.53 (0.85 – 7.55) | 2.58 (0.93 – 7.17) | 2.16 (0.82 – 5.66) |
| RR_4_ | 2.51 (0.84 – 7.51) | 2.48 (0.89 – 6.89) | 2.18 (0.83 – 5.72) |
| 15+ units / week |  |  |  |
| No of deaths | 7 | 16 | 68 |
| RR_1_ | 4.51 (1.43 – 14.2) | 4.69 (1.72 – 12.8) | 7.45 (3.0 – 18.5) |
| RR_2_ | 4.23 (1.34 – 13.4) | 4.54 (1.66 – 12.4) | 6.98 (2.80 – 17.4) |
| RR_3_ | 4.43 (1.40 – 14.0) | 4.60 (1.68 – 12.6) | 6.81 (2.74 – 17.0) |
| RR_4_ | 4.13 (1.30 – 13.0) | 4.44 (1.62 – 12.1) | 6.97 (2.80 – 17.4) |
|  |  |  |  |

| Respiratory deaths |  |  |  |
| --- | --- | --- | --- |
| None |  |  |  |
| No of deaths | 5 | 20 | 59 |
| RR_1_ | 1 | 3.91 (1.47 – 10.4) | 9.15 (3.67 – 22.8) |
| RR_2_ | 1 | 3.62 (1.36 – 9.65) | 6.80 (2.72 – 17.0) |
| RR_3_ | 1 | 3.86 (1.45 – 10.3) | 7.54 (3.02 – 18.8) |
| RR_4_ | 1 | 3.57 (1.34 – 9.51) | 6.77 (2.71 – 16.9) |
| 1-14 units / week |  |  |  |
| No of deaths | 5 | 19 | 65 |
| RR_1_ | 1.70 (0.49 – 5.87) | 3.21 (1.20 – 8.60) | 7.15 (2.88 – 17.8) |
| RR_2_ | 1.76 (0.51 – 6.11) | 3.23 (1.20 – 8.66) | 5.71 (2.30 – 14.2) |
| RR_3_ | 1.79 (0.52 – 6.20) | 3.10 (1.15 – 8.31) | 6.26 (2.52 – 15.6) |
| RR_4_ | 1.73 (0.50 – 5.97) | 3.16 (1.18 – 8.47) | 5.64 (2.27 – 14.0) |
| 15+ units / week |  |  |  |
| No of deaths | 4 | 10 | 77 |
| RR_1_ | 3.12 (0.84 – 11.6) | 3.20 (1.09 – 9.37) | 11.6 (4.68 – 28.7) |
| RR_2_ | 3.84 (1.03 – 14.4) | 3.26 (1.11 – 9.55) | 8.28 (3.33 – 20.6) |
| RR_3_ | 3.10 (0.83 – 11.6) | 3.12 (1.06 – 9.13) | 9.13 (3.68 – 22.7) |
| RR_4_ | 3.79 (1.01 – 14.2) | 3.20 (1.09 – 9.39) | 8.25 (3.32 – 20.5) |
|  |  |  |  |
| Smoking-related cancer deaths |  |  |  |
| None |  |  |  |
| No of deaths | 15 | 34 | 111 |
| RR_1_ | 1 | 2.31 (1.26 – 4.25) | 5.10 (2.97 – 8.74) |
| RR_2_ | 1 | 2.31 (1.26 – 4.24) | 4.83 (2.81 – 8.30) |
| RR_3_ | 1 | 2.31 (1.26 – 4.24) | 4.85 (2.82 – 8.32) |
| RR_4_ | 1 | 2.30 (1.25 – 4.23) | 4.82 (2.81 – 8.28) |
| 1-14 units / week |  |  |  |
| No of deaths | 8 | 39 | 152 |
| RR_1_ | 0.81 (0.34 – 1.91) | 2.26 (1.25 – 4.10) | 4.86 (2.86 – 8.26) |
| RR_2_ | 0.81 (0.34 – 1.92) | 2.32 (1.28 – 4.21) | 4.79 (2.82 – 8.16) |
| RR_3_ | 0.81 (0.35 – 1.92) | 2.32 (1.28 – 4.21) | 4.81 (2.83 – 8.18) |
| RR_4_ | 0.81 (0.34 – 1.92) | 2.29 (1.26 – 4.16) | 4.77 (2.80 – 8.12) |
| 15+ units / week |  |  |  |
| No of deaths | 7 | 24 | 180 |
| RR_1_ | 1.60 (0.65 – 3.92) | 2.36 (1.24 – 4.49) | 7.10 (4.19 – 12.0) |
| RR_2_ | 1.55 (0.63 – 3.80) | 2.33 (1.22 – 4.44) | 6.55 (3.86 – 11.1) |
| RR_3_ | 1.54 (0.63 – 3.78) | 2.32 (1.22 – 4.43) | 6.57 (3.87 – 11.2) |
| RR_4_ | 1.54 (0.62 – 3.77) | 2.31 (1.21 – 4.41) | 6.56 (3.86 – 11.1) |
|  |  |  |  |

* 1 is reference, 95% confidence intervals in parentheses

1 unit =1 measure of spirits, or half a pint of beer or a sixth of a bottle of wine

RR_1_ Relative rate adjusted for age

RR_2_ Relative rate adjusted for age, cholesterol, body mass index, % predicted FEV1, father’s social class, angina, ECG ischaemia and bronchitis

RR_3_ As RR_2_ but excluding body mass index

RR4 As RR_2_ but excluding cholesterol

**Table S2. Number and percentage of men by smoking and alcohol consumption category, excluding deaths in 1^st^ 5 years**

|  |  | Smoking status |  |
| --- | --- | --- | --- |
| Drinking status | Never | Ex | Current |
|  |  |  |  |
| None | 479 (8.7%) | 453 (8.2%) | 822 (14.9%) |
| 1-14 units/week | 354 (6.4%) | 532 (9.6%) | 1217 (22.0%) |
| 15+ units /week | 159 (2.9%) | 363 (6.6%) | 1145 (20.7%) |
|  |  |  |  |

# Table S3. Relative rates of mortality in 30 years of follow-up in men by smoking and alcohol consumption category, excluding deaths in 1^st^ 5 years

|  |  | Smoking status |  |
| --- | --- | --- | --- |
| Drinking status | Never | Ex | Current |
|  |  |  |  |
| All cause deaths |  |  |  |
| None |  |  |  |
| No of deaths | 171 | 240 | 492 |
| RR_1_ | 1* | 1.43 (1.17 – 1.74) | 2.07 (1.74 – 2.47) |
| RR_2_ | 1 | 1.39 (1.14 – 1.69) | 1.97 (1.65 – 2.35) |
| 1-14 units / week |  |  |  |
| No of deaths | 94 | 239 | 656 |
| RR_1_ | 0.86 (0.67 – 1.10) | 1.21 (0.99 – 1.47) | 1.93 (1.63 – 2.28) |
| RR_2_ | 0.86 (0.67 – 1.10) | 1.16 (0.95 – 1.42) | 1.89 (1.59 – 2.24) |
| 15+ units / week |  |  |  |
| No of deaths | 55 | 186 | 720 |
| RR_1_ | 1.13 (0.84 – 1.54) | 1.65 (1.34 – 2.03) | 2.71 (2.29 – 3.20) |
| RR_2_ | 1.04 (0.76 – 1.41) | 1.54 (1.25 – 1.90) | 2.51 (2.12 – 2.97) |
|  |  |  |  |
| Coronary heart disease deaths |  |  |  |
| None |  |  |  |
| No of deaths | 70 | 85 | 176 |
| RR_1_ | 1 | 1.23 (0.89 – 1.69) | 1.75 (1.32 – 2.30) |
| RR_2_ | 1 | 1.16 (0.84 – 1.59) | 1.68 (1.27 – 2.22) |
| 1-14 units / week |  |  |  |
| No of deaths | 34 | 81 | 230 |
| RR_1_ | 0.75 (0.50 – 1.13) | 1.0 (0.73 – 1.38) | 1.59 (1.22 – 2.08) |
| RR_2_ | 0.74 (0.49 – 1.12) | 0.92 (0.67 – 1.27) | 1.58 (1.21 – 2.07) |
| 15+ units / week |  |  |  |
| No of deaths | 14 | 62 | 225 |
| RR_1_ | 0.69 (0.39 – 1.23) | 1.31 (0.93 – 1.85) | 1.95 (1.49 – 2.55) |
| RR_2_ | 0.58 (0.33 – 1.04) | 1.15 (0.82 – 1.62) | 1.83 (1.39 – 2.40) |
|  |  |  |  |
| Stroke deaths |  |  |  |
| None |  |  |  |
| No of deaths | 15 | 20 | 45 |
| RR_1_ | 1 | 1.31 (0.67 – 2.55) | 2.35 (1.31 – 4.21) |
| RR_2_ | 1 | 1.28 (0.65 – 2.50) | 2.34 (1.30 – 4.21) |
| 1-14 units / week |  |  |  |
| No of deaths | 13 | 22 | 62 |
| RR_1_ | 1.47 (0.70 – 3.08) | 1.24 (0.65 – 2.40) | 2.29 (1.30 – 4.03) |
| RR_2_ | 1.49 (0.71 – 3.13) | 1.27 (0.66 – 2.45) | 2.38 (1.35 – 4.20) |
| 15+ units / week |  |  |  |
| No of deaths | 9 | 20 | 70 |
| RR_1_ | 2.33 (1.02 – 5.33) | 2.15 (1.10 – 4.20) | 3.55 (2.03 – 6.22) |
| RR_2_ | 2.27 (0.99 – 5.21) | 2.09 (1.07 – 4.10) | 3.46 (1.97 – 6.09) |
|  |  |  |  |
| Alcohol-related deaths |  |  |  |
| None |  |  |  |
| No of deaths | 4 | 8 | 12 |
| RR_1_ | 1 | 2.15 (0.65 – 7.16) | 2.09 (0.67 – 6.49) |
| RR_2_ | 1 | 2.16 (0.65 – 7.20) | 2.02 (0.65 – 6.27) |
| 1-14 units / week |  |  |  |
| No of deaths | 7 | 13 | 22 |
| RR_1_ | 2.51 (0.73 – 8.58) | 2.91 (0.95 – 8.93) | 2.62 (0.90 – 7.59) |
| RR_2_ | 2.52 (0.74 – 8.64) | 3.02 (0.98 – 9.28) | 2.62 (0.90 – 7.61) |
| 15+ units / week |  |  |  |
| No of deaths | 6 | 14 | 61 |
| RR_1_ | 4.90 (1.38 – 17.4) | 5.22 (1.72 – 15.9) | 8.76 (3.18 – 24.1) |
| RR_2_ | 4.66 (1.31 – 16.6) | 5.11 (1.68 – 15.5) | 8.21 (2.97 – 22.7) |
|  |  |  |  |
| Respiratory deaths |  |  |  |
| None |  |  |  |
| No of deaths | 5 | 19 | 57 |
| RR_1_ | 1 | 3.73 (1.39 – 9.98) | 8.90 (3.57 – 22.2) |
| RR_2_ | 1 | 3.46 (1.29 – 9.28) | 6.68 (2.67 – 16.7) |
| 1-14 units / week |  |  |  |
| No of deaths | 5 | 19 | 64 |
| RR_1_ | 1.70 (0.49 – 5.87) | 3.21 (1.20 – 8.60) | 7.09 (2.85 – 17.6) |
| RR_2_ | 1.77 (0.51 – 6.13) | 3.24 (1.21 – 8.69) | 5.72 (2.30 – 14.3) |
| 15+ units / week |  |  |  |
| No of deaths | 4 | 10 | 76 |
| RR_1_ | 3.12 (0.84 – 11.6) | 3.22 (1.10 – 9.42) | 11.6 (4.67 – 28.6) |
| RR_2_ | 3.80 (1.01 – 14.2) | 3.29 (1.12 – 9.66) | 8.34 (3.35 – 20.8) |
|  |  |  |  |
| Smoking-related cancer deaths |  |  |  |
| None |  |  |  |
| No of deaths | 15 | 31 | 102 |
| RR_1_ | 1 | 2.16 (1.16 – 4.0) | 4.79 (2.79 – 8.24) |
| RR_2_ | 1 | 2.15 (1.16 – 3.99) | 4.59 (2.67 – 7.91) |
| 1-14 units / week |  |  |  |
| No of deaths | 8 | 35 | 142 |
| RR_1_ | 0.80 (0.34 – 1.88) | 2.05 (1.12 – 3.75) | 4.61 (2.71 – 7.84) |
| RR_2_ | 0.80 (0.34 – 1.89) | 2.10 (1.14 – 3.84) | 4.60 (2.70 – 7.85) |
| 15+ units / week |  |  |  |
| No of deaths | 7 | 20 | 160 |
| RR_1_ | 1.58 (0.64 – 3.88) | 2.0 (1.02 – 3.90) | 6.44 (3.79 – 10.9) |
| RR_2_ | 1.50 (0.61 – 3.70) | 1.96 (1.0 – 3.82) | 6.0 (3.53 – 10.2) |
|  |  |  |  |

* 1 is reference, 95% confidence intervals in parentheses

1 unit =1 measure of spirits, or half a pint of beer or a sixth of a bottle of wine

RR_1_ Relative rate adjusted for age

RR_2_ Relative rate adjusted for age, cholesterol, body mass index, % predicted FEV1,

father’s social class, angina, ECG ischaemia and bronchitis

**Table S4. Number of men by smoking and alcohol consumption category, for current and ex-smokers only***

|  | Smoking status | |
| --- | --- | --- |
| Drinking status | Ex | Current |
|  |  |  |
| None | 430 | 857 |
| 1-14 units/week | 513 | 1232 |
| 15+ units /week | 374 | 1191 |
|  |  |  |

*excludes men with missing data on inhalation and age starting and stopping smoking

**Table S5. Relative rates of mortality by smoking and alcohol consumption category in men for current and ex-smokers only***

|  | Smoking status | |
| --- | --- | --- |
| Drinking status | Ex | Current |
|  |  |  |
| All cause deaths |  |  |
| None |  |  |
| No of deaths | 240 | 532 |
| RR_1_ | 1** | 1.44 (1.24 – 1.68) |
| RR_2_ | 1 | 1.42 (1.21 – 1.65) |
| RR_3_ | 1 | 1.45 (1.19 – 1.77) |
| 1-14 units / week |  |  |
| No of deaths | 239 | 690 |
| RR_1_ | 0.84 (0.70 – 1.0) | 1.33 (1.15 – 1.55) |
| RR_2_ | 0.84 (0.70 – 1.01) | 1.35 (1.16 – 1.56) |
| RR_3_ | 0.84 (0.70 – 1.01) | 1.37 (1.13 – 1.67) |
| 15+ units / week |  |  |
| No of deaths | 200 | 777 |
| RR_1_ | 1.14 (0.95 – 1.38) | 1.87 (1.61 – 2.16) |
| RR_2_ | 1.12 (0.93 – 1.35) | 1.79 (1.54 – 2.07) |
| RR_3_ | 1.09 (0.90 – 1.32) | 1.80 (1.48 – 2.18) |
|  |  |  |
| Coronary heart disease deaths |  |  |
| None |  |  |
| No of deaths | 87 | 190 |
| RR_1_ | 1 | 1.38 (1.07 – 1.78) |
| RR_2_ | 1 | 1.43 (1.11 – 1.85) |
| RR_3_ | 1 | 1.46 (1.05 – 2.04) |
| 1-14 units / week |  |  |
| No of deaths | 78 | 252 |
| RR_1_ | 0.76 (0.56 – 1.03) | 1.31 (1.02 – 1.67) |
| RR_2_ | 0.76 (0.56 – 1.03) | 1.41 (1.10 – 1.80) |
| RR_3_ | 0.76 (0.56 – 1.03) | 1.43 (1.03 – 1.98) |
| 15+ units / week |  |  |
| No of deaths | 68 | 248 |
| RR_1_ | 1.05 (0.77 – 1.45) | 1.56 (1.22 – 1.99) |
| RR_2_ | 1.0 (0.72 – 1.37) | 1.58 (1.23 – 2.03) |
| RR_3_ | 0.98 (0.71 – 1.35) | 1.58 (1.14 – 2.19) |
|  |  |  |
| Stroke deaths |  |  |
| None |  |  |
| No of deaths | 21 | 48 |
| RR_1_ | 1 | 1.68 (1.0 – 2.80) |
| RR_2_ | 1 | 1.68 (1.0 – 2.82) |
| RR_3_ | 1 | 1.58 (0.82 – 3.06) |
| 1-14 units / week |  |  |
| No of deaths | 21 | 65 |
| RR_1_ | 0.87 (0.47 – 1.59) | 1.65 (1.0 – 2.70) |
| RR_2_ | 0.91 (0.49 – 1.66) | 1.72 (1.05 – 2.83) |
| RR_3_ | 0.91 (0.49 – 1.66) | 1.62 (0.85 – 3.08) |
| 15+ units / week |  |  |
| No of deaths | 21 | 75 |
| RR_1_ | 1.51 (0.82 – 2.77) | 2.53 (1.55 – 4.12) |
| RR_2_ | 1.53 (0.83 – 2.81) | 2.49 (1.52 – 4.06) |
| RR_3_ | 1.49 (0.81 – 2.75) | 2.34 (1.24 – 4.43) |
|  |  |  |

| Alcohol-related deaths |  |  |
| --- | --- | --- |
| None |  |  |
| No of deaths | 8 | 16 |
| RR_1_ | 1 | 1.17 (0.50 – 2.75) |
| RR_2_ | 1 | 1.16 (0.50 – 2.73) |
| RR_3_ | 1 | 1.25 (0.45 – 3.46) |
| 1-14 units / week |  |  |
| No of deaths | 14 | 24 |
| RR_1_ | 1.42 (0.60 – 3.40) | 1.22 (0.55 – 2.73) |
| RR_2_ | 1.45 (0.61 – 3.47) | 1.24 (0.55 – 2.77) |
| RR_3_ | 1.45 (0.61 – 3.48) | 1.33 (0.50 – 3.52) |
| 15+ units / week |  |  |
| No of deaths | 16 | 68 |
| RR_1_ | 2.52 (1.08 – 5.88) | 4.03 (1.93 – 8.44) |
| RR_2_ | 2.45 (1.05 – 5.74) | 3.91 (1.86 – 8.22) |
| RR_3_ | 2.45 (1.03 – 5.78) | 4.09 (1.63 – 10.3) |
|  |  |  |
| Respiratory deaths |  |  |
| None |  |  |
| No of deaths | 19 | 58 |
| RR_1_ | 1 | 2.24 (1.33 – 3.77) |
| RR_2_ | 1 | 1.85 (1.10 – 3.12) |
| RR_3_ | 1 | 2.76 (1.35 – 5.63) |
| 1-14 units / week |  |  |
| No of deaths | 19 | 63 |
| RR_1_ | 0.85 (0.45 – 1.61) | 1.76 (1.05 – 2.94) |
| RR_2_ | 0.95 (0.50 - 1.80) | 1.53 (0.91 – 2.57) |
| RR_3_ | 0.93 (0.49 – 1.77) | 2.26 (1.11 – 4.59) |
| 15+ units / week |  |  |
| No of deaths | 9 | 77 |
| RR_1_ | 0.71 (0.32 – 1.58) | 2.88 (1.73 – 4.78) |
| RR_2_ | 0.80 (0.36 – 1.78) | 2.28 (1.36 – 3.80) |
| RR_3_ | 0.81 (0.36 – 1.80) | 3.30 (1.64 – 6.67) |
|  |  |  |
| Smoking-related cancer deaths |  |  |
| None |  |  |
| No of deaths | 33 | 111 |
| RR_1_ | 1 | 2.10 (1.42 – 3.10) |
| RR_2_ | 1 | 2.0 (1.35 – 2.96) |
| RR_3_ | 1 | 2.10 (1.26 – 3.51) |
| 1-14 units / week |  |  |
| No of deaths | 39 | 149 |
| RR_1_ | 0.99 (0.62 – 1.57) | 2.0 (1.37 – 2.92) |
| RR_2_ | 1.01 (0.64 – 1.61) | 1.97 (1.34 – 2.87) |
| RR_3_ | 1.01 (0.63 – 1.60) | 2.04 (1.23 – 3.39) |
| 15+ units / week |  |  |
| No of deaths | 24 | 177 |
| RR_1_ | 0.97 (0.57 – 1.64) | 2.87 (1.97 – 4.18) |
| RR_2_ | 0.97 (0.57 – 1.65) | 2.67 (1.83 – 3.89) |
| RR_3_ | 0.91 (0.53 – 1.55) | 2.66 (1.61 – 4.39) |
|  |  |  |

* 30 years of follow-up. Excludes men with missing data on inhalation and age starting and stopping smoking

** 1 is reference, 95% confidence intervals in parentheses

1 unit =1 measure of spirits, or half a pint of beer or a sixth of a bottle of wine

RR_1_ Relative rate adjusted for age

RR_2_ Relative rate adjusted for age, cholesterol, body mass index, % predicted FEV1, father’s social class, angina, ECG ischaemia and bronchitis

RR_3_ Relative rate adjusted for risk factors in RR_2_ and cigarettes/day, inhalation, age started smoking and years smoked to screening.
